# Comparison between first and second wave among critically ill COVID-19 patients admitted to a French ICU: no prognostic improvement during the second wave?

**DOI:** 10.1186/s13054-020-03449-6

**Published:** 2021-01-04

**Authors:** Damien Contou, Megan Fraissé, Olivier Pajot, Jo-Anna Tirolien, Hervé Mentec, Gaëtan Plantefève

**Affiliations:** Service de Réanimation Polyvalente, Centre Hospitalier Victor Dupouy, 69, rue du Lieutenant-Colonel Prud’hon, 95100 Argenteuil, France

**Keywords:** SARS-CoV-2, COVID-19, France, Corticoids, ICU, Mortality, ARDS

As many countries in Europe, France faced a second wave COVID-19 pandemic since September, 2020. During the first wave, intensivists faced an unprecedented massive admission of patients with COVID-19 pneumonia requiring invasive mechanical ventilation, sometimes leading to ICUs saturation. They discovered the stereotypical course of this previously unknown disease with its own specificities including the need for deep sedation and neuromuscular blockade, the increased risk of thrombotic and hemorrhagic events [1, 2], and the prolonged duration of mechanical ventilation [3] with high rate of delirium [4]. Importantly, several randomized controlled trials conducted during this first wave highlighted the beneficial effects of early administration of glucocorticoids for critically ill COVID-19 patients [5, 6].

One can legitimately assume that the experience gained during the first wave may have contributed to a better management and outcome among critically ill COVID-19 patients admitted during the second wave.

We therefore compared the characteristics and the outcome between patients admitted to our 41-bed COVID-19 ICU for acute respiratory failure due to COVID-19 (RT-PCR positive for SARS-CoV-2) during the first wave (from March 13th to May 27th, 2020) and those admitted to our 18-bed ICU during the second wave (from August 19th to December 7th, 2020).

COVID-19 patients without acute respiratory failure, those transferred to other ICUs or still hospitalized in ICU were not included.

Eighty-two patients were admitted during the first wave and 50 during the second wave. Comparison between first and second wave regarding patients’ characteristics, ICU scores, comorbidities, biological data, administered treatments and outcome is detailed in the Table 1. Patients did not differ between the two waves with similar age, ICU scores and comorbidities. Contrary to the first wave, all the patients admitted during the second wave received early glucocorticoids and intermediate or full-dose thromboprophylaxis at ICU admission.

**Table 1:** Comparison between critically ill patients with SARS-CoV-2 pneumonia admitted during the first (*n* = 82) and the second (*n* = 50) COVID-19 wave

	First wave *n* = 82	Second wave *n* = 50	*p* value
**Patient’s characteristics and ICU scores**			
Male sex	66 (81)	38 (76)	0.70
Age, years	62 [55–70]	65 [61–69]	0.20
SAPS II	33 [24–41]	29 [22–37]	0.23
SOFA	4 [3–7]	4 [3–4]	0.07
**Main comorbidities**			
Obesity (body mass index ≥ 30 kg/m^2^)	37 (46)	22 (44)	0.94
Arterial hypertension	52 (63)	30 (60)	0.84
Diabetes mellitus	35 (43)	22 (44)	1.00
Ischemic cardiopathy	8 (10)	5 (10)	1.00
Cerebro-vascular disease	7 (9)	3 (6)	0.74
Chronic kidney failure	7 (9)	5 (10)	0.77
Chronic respiratory disease	18 (23)	14 (28)	0.62
Immunocompromised status	11 (13)	8 (16)	0.88
**Main delays**			
Days between disease onset and ICU admission	8 [7–12]	10 [8–12]	0.42
> 7 days between disease onset and ICU admission	50 (64)	37 (74)	0.33
Days between hospital admission and ICU admission	2 [1–4]	3 [1–4]	0.30
**Biological data at ICU admission**			
D-dimers (ng/mL)	2510 [1655–9222]	1665 [1060–3372]	0.04
Prothrombin time (%)	85 [74–96]	90 [83–101]	0.08
Fibrinogen (g/L)	7.6 [6.0–8.7]	7.4 [6.2–8.4]	0.75
Platelets count (G/L)	225 [170–287]	267 [199–344]	0.03
**Treatment administered at ICU admission**			
Glucocorticoids^a^	10 (12)	50 (100)	< 0.001
Intermediate or full-dose thromboprophylaxis	46 (57)	50 (100)	< 0.001
Antibiotic therapy for bacterial co-infection at ICU admission	18 (22)	14 (29)	0.52
Antiviral drugs (lopinavir-ritonavir or remdesivir)	0 (0)	0 (0)	1.00
Tocilizumab	0 (0)	0 (0)	1.00
**Outcome in the ICU**			
Invasive mechanical ventilation (IMV)	72 (88)	32 (64)	0.01
Days between ICU admission and IMV	2 [1–3]	4 [2–5]	< 0.001
Days between disease onset and IMV	11 [8–13]	14 [11–17]	0.01
Ventilator associated pneumonia	53/72 (74)	24/32 (75)	0.97
Prone positioning	52/72 (72)	30/32 (94)	0.03
Duration of IMV	19 [10–30]	17 [8–31]	0.60
Renal replacement therapy	24 (29)	12 (27)	0.98
Vasopressor support	52 (63)	25 (53)	0.34
Thrombotic events during ICU stay	34 (42)	8 (17)	0.01
Hemorrhagic events during ICU stay	15 (18)	9 (18)	1.00
Length of ICU stay, days	16 [8–30]	14 [9–30]	0.88
Overall ICU mortality	41 (50)	26 (52)	0.96
ICU mortality among patients requiring IMV	41/72 (57)	24/32 (75)	0.13

Continuous variables are reported as median [Interquartile range] and compared between groups using the Student *t* test. Categorical variables are reported as numbers (percentages) and compared using *χ*^2^ test. A *p* value < 0.05 was considered significant

ICU, Intensive Care Unit; IMV, Invasive Mechanical Ventilation; SAPS2, Simplified Acute Physiology Score; SOFA, Sepsis-related Organ Failure Assessment

^a^According to CAPE COVID [5] (first wave) or RECOVERY [6] (second wave) protocols

Compared to the first wave, we observed a lower proportion of patients requiring invasive mechanical ventilation and a lower rate of thrombotic events. The delay between ICU admission and tracheal intubation was longer during the second wave. ICU mortality (50% vs. 52%, *p* = 0.96) and duration of ICU stay did not differ between the two waves.

The Kaplan–Meier survival analysis did not show significant difference between the two waves (*p* = 0.90, log-rank test) (Fig. 1).

**Fig. 1:**
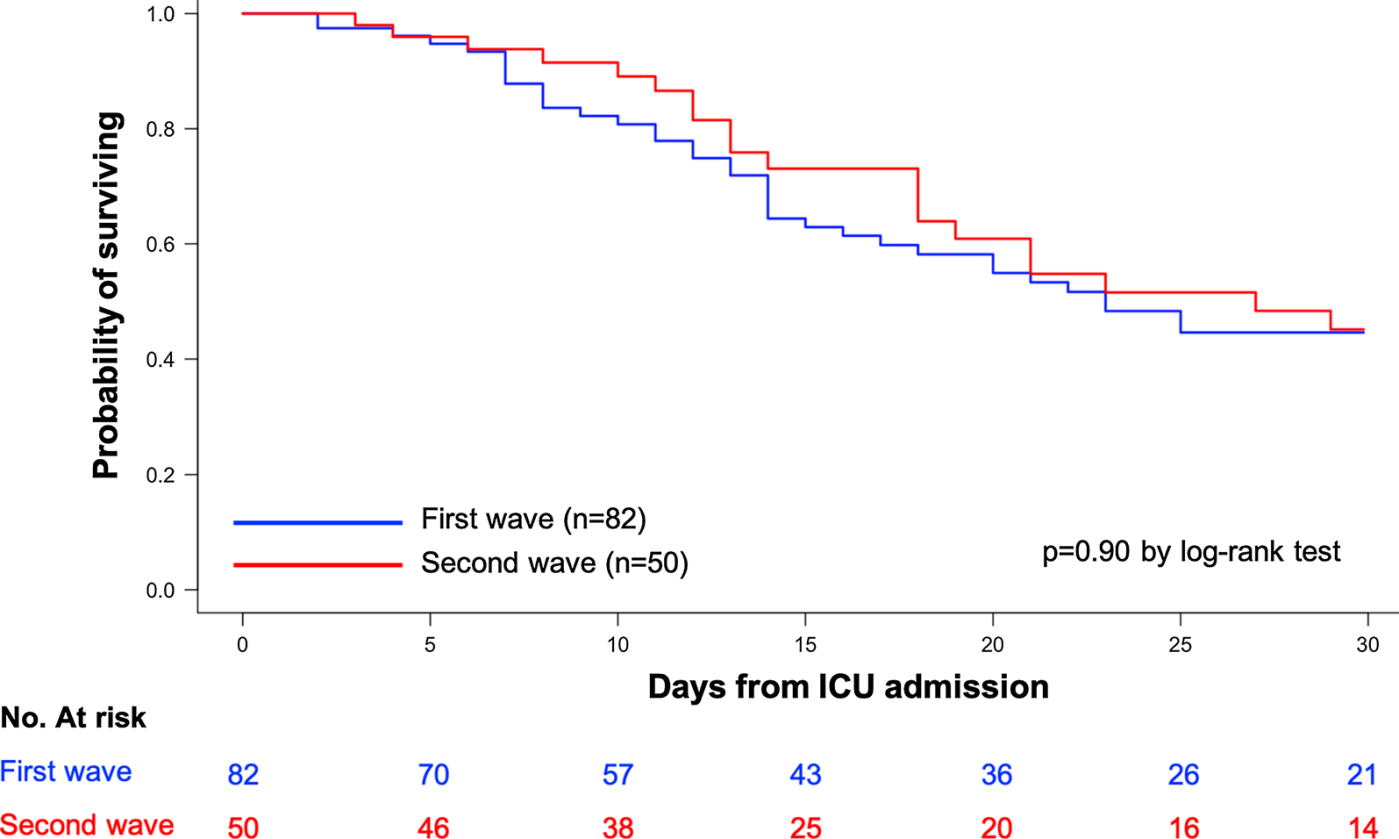
Kaplan–Meier survival estimates during the 30 days following ICU admission of COVID-19 patients admitted during the first wave (blue curve) and the second wave (red curve)

We herein report the first study comparing the outcome of critically ill COVID-19 patients between first and second wave in France. Despite a better understanding of COVID-19 with significant treatment modification including systematic and early administration of glucocorticoids as well as intermediate/full dose thromboprophylaxis, we did not observe any decrease in ICU mortality, with still half of the patients dying in our ICU.

However, patients admitted during the second wave were less likely to require invasive mechanical ventilation with a longer delay between ICU admission and tracheal intubation, potentially related to early administration of glucocorticoids [6]. However, this longer delay questions about the higher, albeit not statistically significant, ICU mortality observed in our intubated patients during the second wave.

The lower rate of thrombotic events observed during the second wave is likely inherent to the increased intensity of thromboprophylaxis even if a lower rate of CT pulmonary angiographies during the second wave cannot be ruled out.

The strengths of our study include the similar typology and severity of the patients during both waves making relevant the comparison. Its monocenter design, implying similar criteria of ICU admission and similar criteria of tracheal intubation by the same team of intensivists during both periods, may also be considered as a strength.

## Data Availability

The dataset used and analyzed for the current study is available from the corresponding author on reasonable request.
